# Deep learning-based early prediction of carotid plaque response to lipid-lowering therapy using longitudinal multimodal ultrasound imaging

**DOI:** 10.1186/s13244-026-02286-5

**Published:** 2026-04-21

**Authors:** Lulu Jiang, Yaning Sun, Wendi Huang, Saisai Wang, Danqin Pan, Yanming Zhang, Mengjie Liang

**Affiliations:** 1 Department of Ultrasound Imaging, The First People’s Hospital of Wenling (Taizhou University Affiliated Wenling Hospital), School of Medicine, Taizhou University, Taizhou, People’s Republic of China; 2 Department of Special Examination, Haining Central Hospital, Haining City, People’s Republic of China; 3 Wenling Institute of Big Data and Artificial Intelligence in Medicine, Taizhou, People’s Republic of China; 4 Faculty of Applied Sciences, Macao Polytechnic University, Macao, People’s Republic of China

**Keywords:** Deep learning, Atherosclerotic plaque, Treatment efficacy, Ultrasound imaging, Longitudinal study

## Abstract

**Objective:**

This study aimed to develop and validate a deep learning prediction model using longitudinal multimodal ultrasound imaging for early identification of treatment-sensitive and treatment-resistant carotid plaques in patients receiving lipid-lowering therapy.

**Materials and methods:**

This prospective study enrolled 802 patients with vulnerable carotid plaques or stenosis ≥ 50%. Patients underwent serial multimodal ultrasound examinations, including B-mode imaging, superb microvascular imaging, and shear wave elastography at baseline and 3, 6, 9, and 12 months after initiating statin therapy. The dataset was divided into training and testing sets using stratified sampling with data augmentation. A hybrid DL model combining convolutional neural networks and long short-term memory networks analyzed longitudinal imaging sequences integrated with baseline clinical data. Five progressive prediction models were constructed for baseline and each follow-up time point, sharing identical architecture but trained independently on temporal sequences of varying lengths using 5-fold cross-validation. Model performance was assessed for discrimination ability, calibration consistency, and clinical utility.

**Results:**

Five progressive prediction models demonstrated characteristic temporal performance patterns, with significant improvement from 3 to 6 months (AUC 0.866), followed by marginal gains. The 6-month model emerged as the most clinically practical assessment time point, achieving high specificity (93.7%) for early therapeutic decisions. Ablation experiments confirmed imaging features as primary predictive determinants, while attention mapping revealed consistent focus on plaque-adjacent regions, validating that treatment response prediction relies on morphological changes within target plaques.

**Conclusion:**

A hybrid DL model enables reliable carotid plaque treatment response prediction within six months, optimizing personalized therapy through earlier identification of treatment-resistant patients.

**Critical relevance:**

This study validates deep learning algorithms to predict carotid plaque treatment response within six months, advancing clinical radiology practice by enabling earlier therapeutic optimization through objective ultrasound-based assessment.

**Key Points:**

- Conventional imaging requires 12 months to reliably assess plaque treatment response.
- Deep learning model predicts treatment response at six months with high accuracy.
- Earlier prediction enables timely therapeutic adjustments for resistant patients.

**Graphical Abstract:**

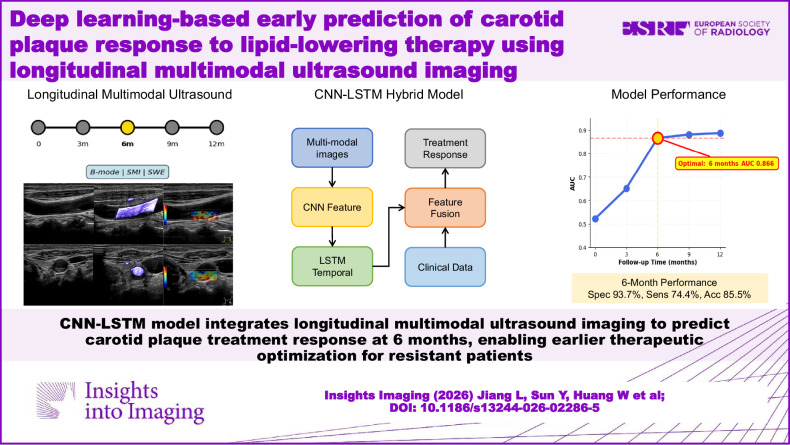

## Introduction

Current clinical guidelines recommend statin therapy for patients with vulnerable carotid plaques or stenosis ≥ 50% [1], with robust evidence supporting cardiovascular protection through lipid-lowering effects and pleiotropic mechanisms [2]. However, individual responses to statin therapy vary considerably, with substantial heterogeneity observed in plaque regression rates and compositional changes among patients receiving similar treatment regimens [3, 4].

Assessment of treatment response is constrained by difficulty in detecting subtle plaque changes, which typically require extended treatment periods before becoming reliably detectable [5]. Although magnetic resonance imaging (MRI) has emerged as the most accurate non-invasive modality for plaque characterization [6, 7], its clinical application for frequent longitudinal assessment is limited by high costs, restricted availability, and practical implementation barriers [8]. This creates a critical clinical gap where poor responders continue suboptimal therapy for extended periods, potentially missing opportunities for timely therapeutic modifications. Vascular ultrasound represents a promising solution, offering superior feasibility for repeated longitudinal assessment [9]. Recent technological advances, including superb microvascular imaging (SMI) and shear wave elastography (SWE), have enhanced ultrasound capabilities to detect morphological alterations in plaque composition, particularly echogenicity changes correlating with plaque stabilization [10–12]. Building upon these developments, we hypothesize that temporal deep learning (DL) algorithms analyzing longitudinal multimodal ultrasound data could enable early prediction of carotid plaque response to lipid-lowering therapy, thereby facilitating timely therapeutic adjustments for treatment-resistant patients.

This study aimed to develop and validate a temporal DL prediction model using longitudinal multimodal ultrasound imaging data for early identification of treatment-sensitive and treatment-resistant carotid plaques within the first few months of lipid-lowering therapy initiation. Such early prediction capability could optimize personalized treatment strategies by enabling timely therapeutic adjustments for poor responders, potentially improving patient outcomes while reducing costs associated with prolonged ineffective treatments.

## Materials and methods

This prospective cohort study was conducted in accordance with the Declaration of Helsinki and received approval from the Institutional Review Boards of the First People’s Hospital of Wenling (KY-2025-2022-01). All participating patients provided written informed consent, and all data were anonymized.

### Patient selection

We evaluated patients with vulnerable carotid plaques or stenosis ≥ 50% who were candidates for lipid-lowering therapy at our institution between January 2022 and June 2024. Among 943 consecutive patients screened, those meeting the following inclusion criteria were selected: (1) age 18–75 years; (2) no history of ischemic stroke or transient ischemic attack; (3) either statin-naïve or discontinued statin therapy for at least 12 months prior to enrollment; (4) ability to undergo serial ultrasound examinations; and (5) willingness to participate in longitudinal follow-up assessments over a minimum period of 12 months. Exclusion criteria included previous carotid endarterectomy or stenting procedures, contraindications to statin therapy, inadequate ultrasound visualization of carotid plaque, and inability to complete required follow-up visits.

### Ultrasound imaging protocol

All patients underwent carotid ultrasound examination using a GE Logiq E20 ultrasound system with a 3–12 MHz linear array transducer. Examinations were performed by two experienced sonographers with over 15 years of specialized training in carotid imaging. Comprehensive documentation included bilateral common carotid arteries, internal carotid arteries, and external carotid arteries using standardized protocols. Three complementary imaging modalities were systematically acquired at each carotid plaque: conventional B-mode grayscale imaging for morphological assessment, SMI for vascular perfusion evaluation, and SWE for tissue stiffness characterization.

For treatment response analysis, target plaques were identified as those requiring therapeutic intervention and longitudinal monitoring, including vulnerable plaques (predominantly hypoechoic or heterogeneous) and plaques causing carotid stenosis ≥ 50% according to North American Symptomatic Carotid Endarterectomy Trial (NASCET) criteria [13]. For patients with multiple qualifying plaques, the most clinically significant plaque was selected based on priority order: the most vulnerable plaque, the plaque causing the highest degree of stenosis, or the largest plaque when other criteria were equivalent. For treatment outcome classification, plaques were categorized as showing a favorable treatment response or poor response at 12-month follow-up. Favorable treatment response was defined as meeting any of the following criteria: reduction in plaque area ≥ 5%, decrease in plaque thickness ≥ 0.4 mm, or favorable echogenicity changes, including increased echogenicity or improved homogeneity. Plaques not meeting these criteria were classified as showing poor response. Each plaque was analyzed independently by both sonographers, and in cases of disagreement, a third senior physician with over 20 years of experience in carotid plaque diagnosis served as an expert adjudicator to provide the final determination.

### Statin therapy and follow-up

All enrolled patients were initiated on standardized medical therapy according to current guidelines for asymptomatic carotid atherosclerosis management [14]. This included high-intensity statin therapy with atorvastatin 20 mg daily (target LDL-C < 1.8 mmol/L), with ezetimibe added if needed. Antiplatelet therapy with aspirin 100 mg daily was prescribed based on individual cardiovascular risk-benefit assessment. Appropriate antihypertensive treatment for patients with hypertension was provided when indicated. Medication adherence was assessed through standardized questionnaires at each follow-up visit. Baseline laboratory evaluation, including lipid profiles, was performed for demographic characterization. Carotid ultrasound follow-up examinations were conducted at 3, 6, 9, and 12 months after therapy initiation using identical imaging protocols. Target plaques were systematically re-evaluated at each visit using identical multimodal protocols to assess morphological, perfusion, and stiffness changes.

### Image processing

For each target plaque, longitudinal and transverse axis views were acquired using three complementary imaging modalities (B-mode, SMI, and SWE) during the same scanning session with fixed transducer positioning. The ultrasound system maintained spatial alignment when switching between modalities, ensuring pixel-level correspondence without requiring additional image registration.

All images were converted to single-channel grayscale representations where B-mode images preserved tissue echogenicity, SMI images represented vascular perfusion intensity, and SWE images quantified tissue stiffness from the color-encoded maps. Images were resized to 224 × 224 pixels using bilinear interpolation and normalized to the 0–1 range independently for each modality.

At each time point, three modality images from the same anatomical plane were concatenated along the channel dimension. Longitudinal and transverse data were further concatenated to generate 6-channel inputs (224 × 224 × 6). This process across five time points (baseline, 3, 6, 9, and 12 months) resulted in 30 images per plaque for complete longitudinal analysis.

### Data preprocessing and augmentation

The complete dataset was constructed from enrolled patients, with each contributing longitudinal ultrasound imaging sequences of their target plaque and baseline clinical variables. This dataset was randomly divided into training and testing sets using stratified sampling with an 8:2 ratio to maintain balanced representation of treatment response outcomes. To enhance model robustness, data augmentation was applied to the training set through geometric transformations of imaging data (rotation ±5°, horizontal flipping, brightness adjustment ±10%), with corresponding clinical features replicated to maintain patient-level correspondence. The testing set remained unaugmented to ensure objective evaluation.

### DL model development

**Fig. 1:**
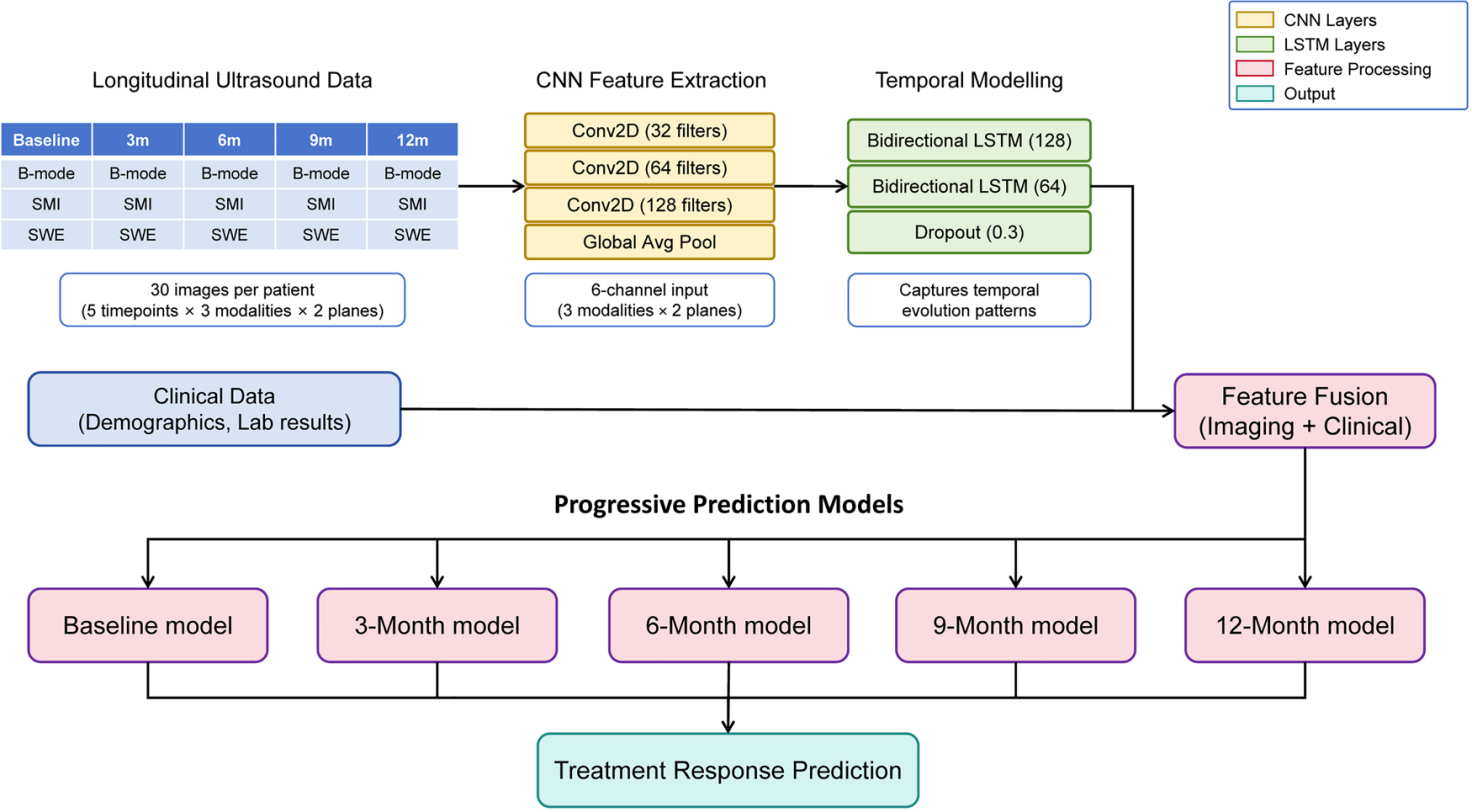
DL model architecture for carotid plaque treatment response prediction. A hybrid CNN-LSTM architecture processing multi-modal longitudinal ultrasound data and clinical features to generate five progressive prediction models for treatment response assessment at baseline, 3, 6, 9, and 12 months

Our DL architecture (Fig. 1) processed longitudinal ultrasound imaging sequences of 30 images per target plaque (5 time points × 6 channels), integrated with baseline clinical data. The model employed a hybrid approach combining convolutional neural networks (CNNs) for spatial feature extraction with long short-term memory (LSTM) networks for temporal pattern recognition.

The CNN feature extractor employed three convolutional layers with filter depths of 32, 64, and 128, respectively. All convolutional layers used 3 × 3 kernels with a stride of 1 and same padding, followed by batch normalization, ReLU activation, and max pooling with 2 × 2 kernels and a stride of 2. This architecture progressively reduced spatial dimensions from 224 × 224 through 112 × 112 and 56 × 56 to 28 × 28 pixels while expanding feature channels from 6 to 128. Global average pooling was applied after the final convolutional layer, followed by fully connected layers with 128 and 64 units and 0.3 dropout, generating a 64-dimensional feature vector at each time point.

For temporal modeling, the CNN processed each time point’s 6-channel ultrasound image independently to generate feature vector sequences. Two bidirectional LSTM layers with 128 and 64 hidden units, respectively, both with 0.3 dropout, processed these temporal sequences. Clinical features demonstrating significant associations with treatment response in univariable analysis were selected for model input. They were processed through separate fully connected layers and concatenated with the LSTM output before final binary classification via sigmoid activation. Five progressive prediction models were constructed for baseline, 3-month, 6-month, 9-month, and 12-month assessments. All models shared an identical CNN-LSTM architecture but were trained independently on temporal sequences of varying lengths. The baseline model processed single-timepoint data, while subsequent models incorporated cumulative data from baseline through their respective evaluation timepoints, with sequence lengths ranging from 1 to 5. Supplementary Fig. S1 illustrates the complete data flow from raw ultrasound images through feature extraction and temporal modeling to final prediction, detailing the specific temporal sequence organization for each progressive model.

Training was conducted using 5-fold stratified cross-validation to comprehensively evaluate performance while maximizing data utilization. Within each fold, models were trained for a maximum of 200 epochs with a batch size of 16. The Adam optimizer was initialized with a learning rate of 0.001, beta1 of 0.9, and beta2 of 0.999, following standard default values widely validated for medical imaging tasks. Learning rate reduction was implemented with a factor of 0.5 when validation loss failed to improve for 15 consecutive epochs, with a minimum learning rate threshold of 1 × 10^−7^. Early stopping was applied with patience of 30 epochs based on validation loss monitoring. The binary cross-entropy loss function was employed for the classification task as the standard objective for binary classification. Model convergence was evaluated using validation loss and accuracy metrics, with final model selection based on the epoch achieving the lowest validation loss before evaluation on the independent testing set. For each time point, the hyperparameters yielding the lowest validation loss across five folds were selected to train the final model on the entire training dataset.

### Model performance evaluation

Model performance was comprehensively evaluated on the independent testing set to assess discrimination ability, calibration performance, and clinical utility. All five progressive prediction models underwent identical evaluation procedures to enable systematic comparison of predictive capability across different time points. Model interpretability analysis was conducted on each model to enhance clinical understanding. Ablation experiments were performed to quantify the relative contributions of imaging features vs clinical features. Attention map analysis was implemented to visualize spatial regions within ultrasound images that most influenced treatment response predictions. Clinical feature importance assessment was conducted to rank the predictive contribution of individual clinical variables.

### Statistical analysis

Continuous variables were assessed for normality using the Shapiro–Wilk test and compared between groups using independent samples *t*-tests or Mann–Whitney *U*-tests as appropriate. Categorical variables were compared using chi-square tests. Model discrimination was evaluated using receiver operating characteristic (ROC) analysis with area under the curve (AUC) calculation, compared using DeLong’s test. Optimal decision thresholds were determined using Youden’s index, and classification metrics including accuracy, sensitivity, specificity, positive and negative predictive values (NPVs) were derived from confusion matrices. Model calibration was assessed through calibration plots and Hosmer–Lemeshow (HL) goodness-of-fit testing. Clinical utility was quantified using decision curve analysis (DCA). All analyses were performed using Python 3.10 with statistical significance set at *p* < 0.05.

## Results

### Patient characteristics

**Fig. 2:**
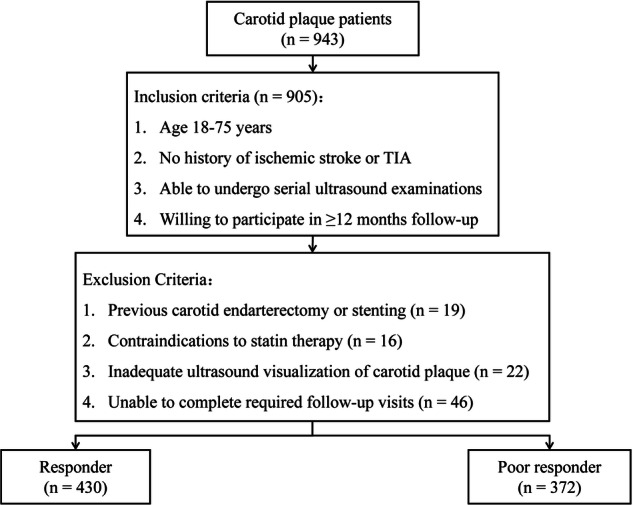
Patient selection flowchart showing the enrollment process from initial screening to the final study cohort

After applying the selection criteria, 802 patients were included in the final analysis cohort for development and validation of the DL prediction model (Fig. 2). Based on the response criteria assessed at 12-month follow-up, the cohort comprised 430 treatment-favorable responders and 372 poor responders. Table 1 presents baseline clinical and ultrasound imaging characteristics stratified by treatment response status. Poor responders were significantly older and demonstrated a higher prevalence of diabetes mellitus and current smoking, along with unfavorable lipid profiles characterized by elevated LDL cholesterol and triglyceride levels and reduced high-density lipoprotein cholesterol levels (all *p* < 0.05). Baseline ultrasound characteristics also differed significantly between groups, with poor responders exhibiting greater plaque thickness, higher stenosis degree, and more heterogeneous composition (all *p* < 0.05).

**Table 1:** Baseline clinical and imaging characteristics of study participants

Characteristic	Favorable responder	Poor responder	*p*-value
	(*n* = 430)	(*n* = 372)	
Clinical characteristics			
Age, years	63 (59, 68)	64 (60, 68)	0.028
Male gender, *n* (%)	244 (56.7)	228 (61.3)	0.192
BMI, kg/m^2^	26.0 ± 3.3	26.2 ± 3.5	0.505
Hypertension, *n* (%)	299 (69.5)	272 (73.1)	0.264
Diabetes mellitus, *n* (%)	132 (30.7)	146 (39.3)	0.011
Current smoking, *n* (%)	126 (29.3)	144 (38.7)	0.005
Family history of CAD, *n* (%)	106 (24.7)	81 (21.8)	0.337
Total cholesterol, mmol/L	5.2 ± 1.1	5.3 ± 1.2	0.167
LDL cholesterol, mmol/L	3.1 ± 0.9	3.3 ± 1.0	0.002
HDL cholesterol, mmol/L	1.3 (1.0, 1.6)	1.2 (1.0, 1.4)	0.002
Triglycerides, mmol/L	1.8 (1.3, 2.3)	2.0 (1.6, 2.7)	< 0.001
Antihypertensive therapy, *n* (%)	271 (63.0)	244 (65.6)	0.449
Previous statin use, *n* (%)	88 (20.5)	90 (24.2)	0.205
Imaging characteristics			
Plaque location, *n* (%)			0.661
Internal carotid artery	144 (33.5)	121 (32.5)	
Common carotid artery	121 (28.1)	97 (26.1)	
Carotid bifurcation	165 (38.4)	154 (41.4)	
Plaque thickness, mm	2.9 (2.3, 3.4)	3.1 (2.5, 3.9)	0.028
Stenosis degree, %	34.9 (27.0, 48.4)	38.4 (28.2, 57.7)	0.016
Echogenicity, *n* (%)			0.394
Hypoechoic	152 (35.4)	131 (35.2)	
Isoechoic	80 (18.6)	56 (15.1)	
Hyperechoic	13 (3.0)	8 (2.2)	
Heterogeneous	185 (43.0)	177 (47.6)	
Homogeneity, *n* (%)			0.004
Homogeneous	179 (41.6)	118 (31.7)	
Heterogeneous	251 (58.4)	254 (68.3)	
Surface characteristics, *n* (%)			0.478
Smooth	266 (61.9)	221 (59.4)	
Irregular	164 (38.1)	151 (40.6)	
Calcification, *n* (%)	268 (62.3)	228 (61.3)	0.763

*BMI* body mass index, *CAD* coronary artery disease, *LDL* low-density lipoprotein, *HDL* high-density lipoprotein

The cohort was divided into training and testing sets using stratified random sampling, with the training set including 637 patients (286 poor responders, 44.9%) and the testing set containing 165 patients (86 poor responders, 52.1%). Baseline characteristics were well-balanced between training and testing sets (all *p*-values > 0.05, Table S1), ensuring appropriate dataset partitioning for model development and validation.

### Model training

Following the data augmentation protocol, the training set was expanded to 2548 patient-level samples, with transformations applied consistently across all temporal sequences to preserve longitudinal coherence. Five progressive prediction models were successfully developed for treatment response assessment at baseline, 3-month, 6-month, 9-month, and 12-month time points using the hybrid CNN-LSTM architecture.

Model development was conducted using 5-fold stratified cross-validation on the augmented training set. In each fold, 2038 samples (80%) were allocated for training and 510 samples (20%) for validation, maintaining balanced representation of treatment response outcomes. The batch size of 16 resulted in approximately 127 iterations per epoch within each validation fold. For each time point, the model configuration achieving the lowest validation loss across all five folds was selected and retrained on the entire augmented training set (2548 samples) to generate the final model for subsequent evaluation.

**Fig. 3:**
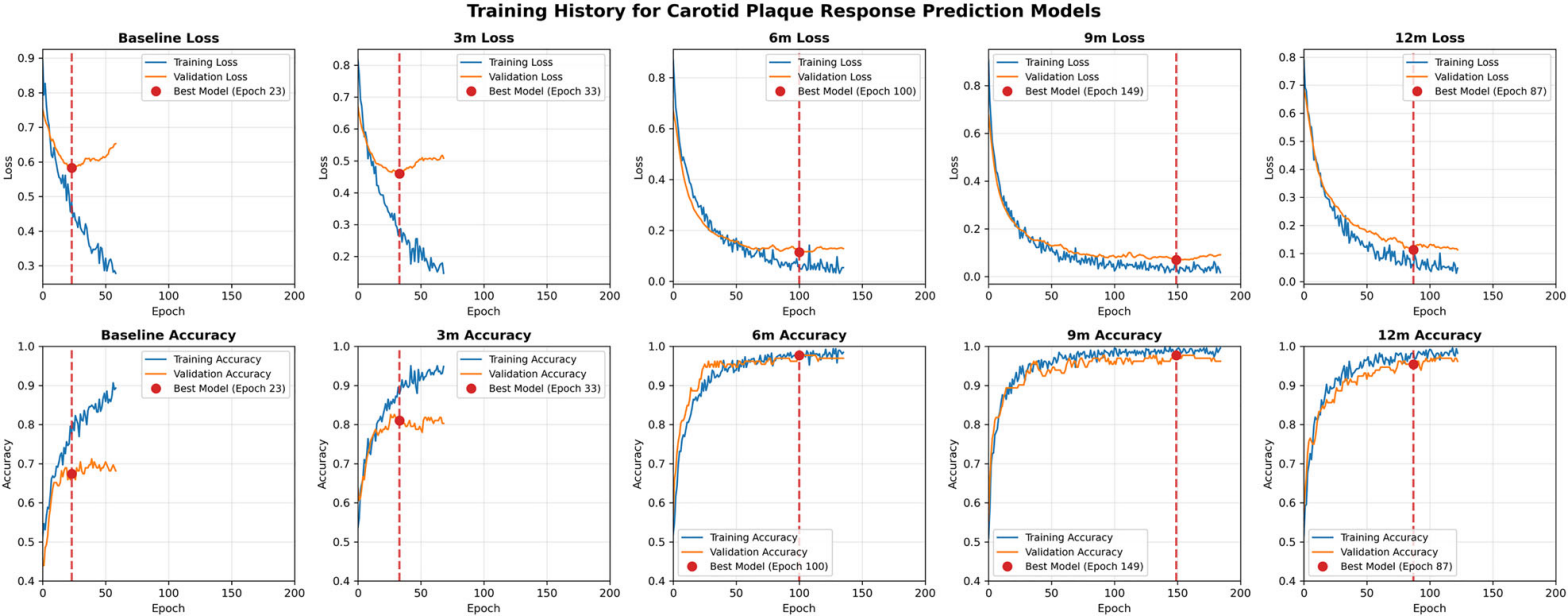
Training history for carotid plaque treatment response prediction models at different time points

Figure 3 illustrates the training history for these final models, demonstrating stable convergence patterns without overfitting across all prediction time points. All models achieved convergence with consistent alignment between training and validation performance curves, indicating robust learning capability. The training process showed progressive improvement in model performance with longer follow-up periods, as evidenced by enhanced validation accuracy and reduced validation loss. Detailed training parameters and convergence metrics for each model are provided in Table S2.

### Model performance evaluation

**Fig. 4:**
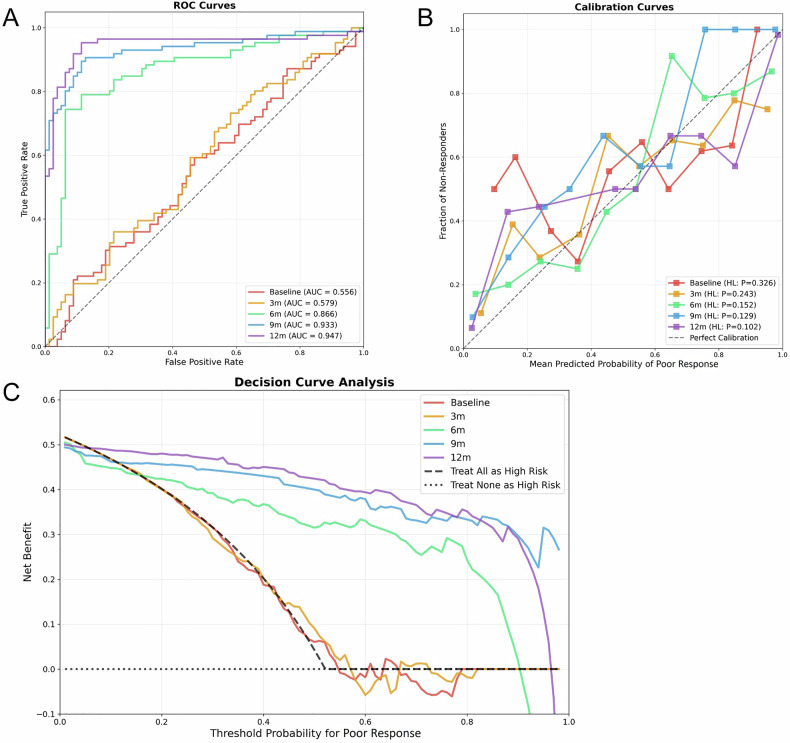
Model performance evaluation across different time points. **A** ROC curve showing the discriminative performance of the five progressive prediction models. **B** Calibration plots demonstrating agreement between predicted probabilities and observed outcomes. **C** DCA illustrating clinical utility and net benefit across threshold probabilities

The progressive prediction models demonstrated a characteristic temporal performance pattern as illustrated in Fig. 4 and Table 2, with 6 months identified as the optimal assessment time point. Early prediction models showed limited discriminative capability, with baseline and 3-month assessments clustering near the diagonal reference line in ROC analysis. The DeLong test revealed a statistically significant improvement from 3 months to 6 months (AUC = 0.866, *p*-values < 0.001). Subsequently, performance stabilized with no significant differences between 6-month, 9-month, and 12-month models (all *p*-values > 0.05). Calibration assessment using the HL test demonstrated excellent agreement for models beyond 3 months, with calibration plots showing progressively improved concordance. DCA showed diminishing marginal returns beyond 6 months, with the substantial performance gains between 3 and 6 months exceeding incremental improvements from extended follow-up.

**Table 2:** Performance metrics of progressive prediction models for carotid plaque treatment response assessment

Model	Threshold	Accuracy	Sensitivity	Specificity	PPV	NPV	Precision	Recall	F1_score	TP	TN	FP	FN
Baseline	0.517	0.564	0.593	0.532	0.580	0.545	0.580	0.593	0.586	51	42	37	35
3 m	0.502	0.582	0.733	0.418	0.578	0.589	0.578	0.733	0.646	63	33	46	23
6 m	0.597	0.836	0.744	0.937	0.928	0.771	0.928	0.744	0.826	64	74	5	22
9 m	0.430	0.891	0.895	0.886	0.895	0.886	0.895	0.895	0.895	77	70	9	9
12 m	0.524	0.921	0.953	0.886	0.901	0.946	0.901	0.953	0.927	82	70	9	4

*PPV* positive predictive value, *NPV* negative predictive value, *TP* true positive, *TN* true negative, *FP* false positive, *FN* false negative

The 6-month model achieved high specificity with excellent positive predictive value (PPV), corresponding to only 5 false positives (FPs) in the testing cohort. While sensitivity remained at 0.744 with 22 false negatives (FNs), this pattern minimizes misclassification of poor responders as treatment-sensitive while maintaining acceptable detection rates. FP predictions would lead to unnecessary treatment modifications, whereas FNs can be addressed through subsequent assessment. The 9-month model demonstrated further improvement with substantially reduced FN rates. These findings support a staged implementation strategy where 6-month positive predictions trigger immediate therapeutic interventions, while negative predictions warrant reassessment at 9 months.

### Model interpretability analysis

Comprehensive interpretability analysis was conducted across the 6-month, 9-month, and 12-month prediction models, comprising quantitative feature importance assessment and spatial attention mapping. Ablation study was performed to quantify the relative contributions of imaging and clinical features to model predictive performance (Table 3). The comparison revealed that imaging-only models achieved performance levels closely approximating the full hybrid models across all time points, while clinical-only models demonstrated substantially inferior and relatively stable performance. This pattern confirms the dominant role of longitudinal imaging features in treatment response prediction, with clinical features providing supplementary rather than primary discriminative information.

**Table 3:** Ablation study demonstrating relative contributions of imaging and clinical features to treatment response prediction performance across temporal models

Time point	Full model AUC	Imaging-only AUC	Clinical-only AUC
Baseline	0.556	0.520	0.523
3 m	0.579	0.545	0.525
6 m	0.866	0.820	0.532
9 m	0.933	0.898	0.540
12 m	0.947	0.920	0.548

**Fig. 5:**
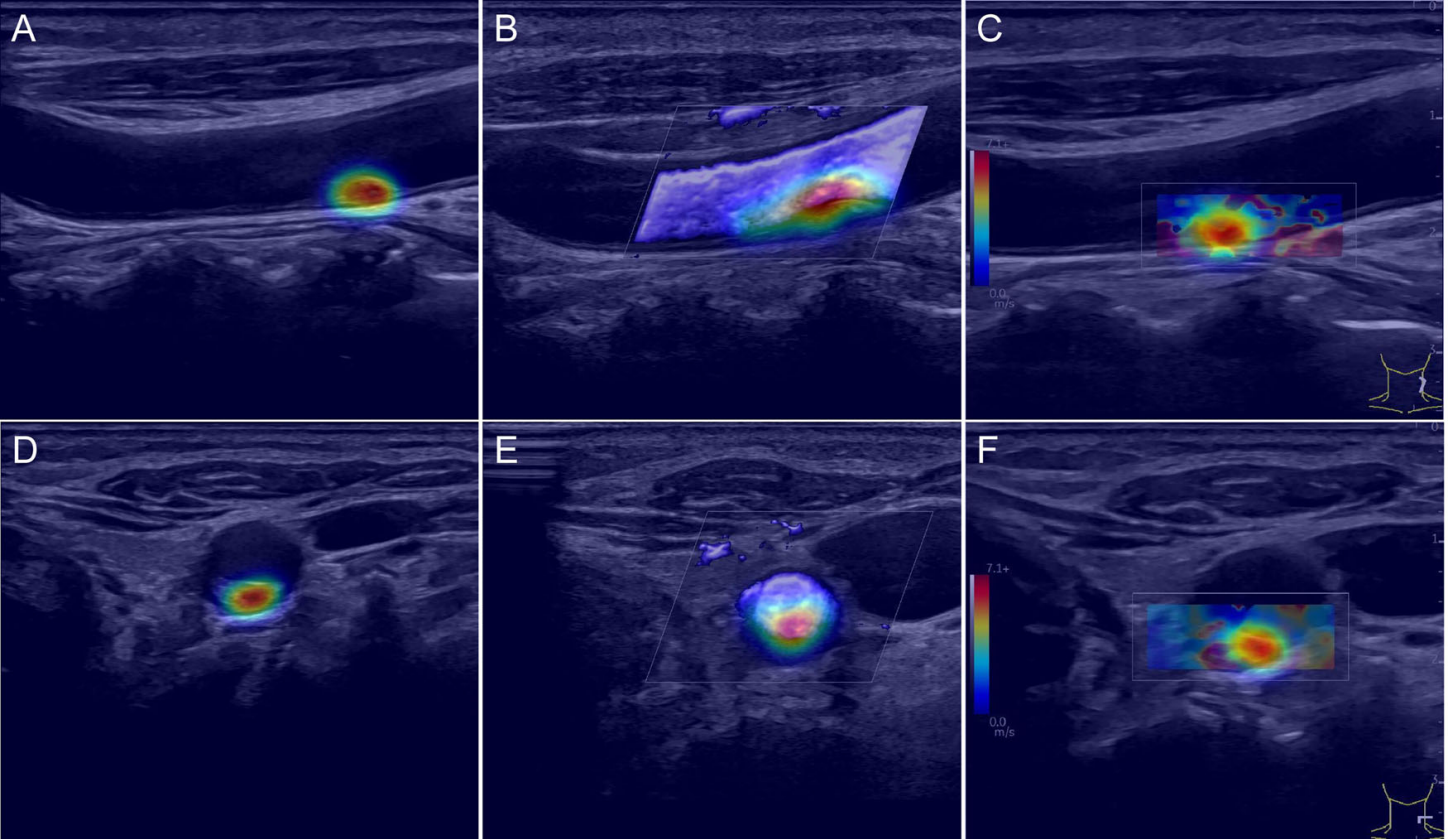
Attention maps for a 6-month prediction model across multimodal ultrasound imaging. (**A**) Longitudinal B-mode, (**B**) longitudinal SMI, (**C**) longitudinal SWE, (**D**) transverse B-mode, (**E**) transverse SMI, and (**F**) transverse SWE imaging. Attention intensity patterns vary across different modalities and acquisition planes, with high-intensity regions indicating areas contributing most to treatment response prediction

Building upon this finding of imaging dominance, attention map analysis was conducted to identify the specific spatial regions that contributed most to the imaging-based predictions. Figure 5 presents a representative example of attention maps across multimodal ultrasound imaging, demonstrating varying attention intensity patterns across different modalities and acquisition planes. High-intensity attention regions indicate areas contributing most significantly to treatment response prediction, with the model showing selective focus on clinically relevant anatomical features within the target plaque region.

**Fig. 6:**
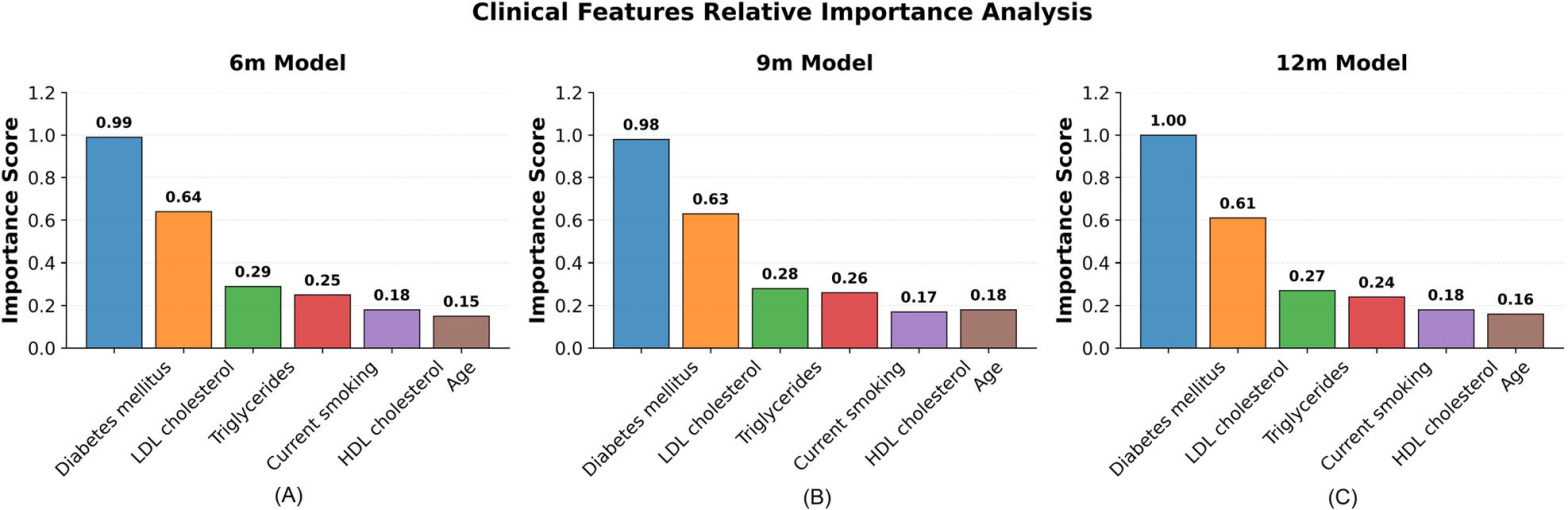
Clinical features relative importance analysis across temporal prediction models. Importance scores for individual clinical variables in (**A**) 6-month, (**B**) 9-month, and (**C**) 12-month models, demonstrating consistent patterns with diabetes mellitus as the dominant predictor and stable feature importance distribution across time points

Complementing the imaging analysis, clinical feature importance assessment (Fig. 6) was performed to characterize the supplementary role of clinical variables identified in the ablation study. The analysis demonstrated that diabetes mellitus consistently emerged as the predominant clinical predictor across all temporal models, while other clinical variables showed relatively modest contribution levels. The hierarchical importance pattern remained stable from 6 to 12 months, confirming the complementary role of clinical features within the hybrid model architecture and supporting the ablation study findings.

## Discussion

This study developed and validated a hybrid DL model integrating longitudinal multimodal ultrasound imaging with clinical data for carotid plaque treatment response prediction. The six-month model achieved optimal clinical performance with high specificity and robust accuracy. This represents the first validated evidence that DL combined with conventional ultrasound can advance statin therapy response assessment to six months, enabling earlier identification of treatment-resistant populations and timely therapeutic adjustments that may improve patient outcomes while reducing costs associated with prolonged ineffective treatments.

### DL advantages

For patients with vulnerable carotid plaques or severe stenosis, statin therapy represents the cornerstone of medical management, yet individual responses demonstrate substantial heterogeneity [2, 6, 15]. Conventional imaging assessment remains constrained by the inability to detect subtle morphological changes during early treatment phases, with statins achieving significant reductions in lipid-rich necrotic core volume only after 12 months of therapy [4, 16]. DL technologies can overcome these limitations by identifying patterns imperceptible to human visual assessment [17, 18] and detecting minute morphological alterations within complex imaging data, offering advantages in scalability, reproducibility, and rapid technological advancement [19]. These capabilities enable predictive models that can revolutionize early treatment response assessment, providing clinicians with tools for personalized therapeutic decision-making in carotid atherosclerosis management [20].

### Temporal DL architecture and performance

Our hybrid CNN-LSTM architecture leverages the complementary strengths of spatial feature extraction and temporal sequence modeling. The CNN component captures morphological features, including spatial hierarchies and structural patterns, while LSTM networks characterize sequential dependencies and temporal interactions within longitudinal imaging data [21, 22]. Evaluation across five progressive temporal models demonstrated the effectiveness of this architectural design. The six-month model achieved robust discrimination between favorable responders and poor responders, with the marked improvement from three months to six months confirming that our temporal architecture successfully captures the critical window where treatment-induced changes accumulate to predictive thresholds.

This performance characteristic distinguishes our approach from existing artificial intelligence (AI) applications in plaque imaging. Recent studies have demonstrated AI success in automated plaque quantification and characterization, with DL models achieving high accuracy for carotid plaque segmentation from ultrasound [23, 24]. However, these approaches have predominantly focused on single-time-point plaque detection and cardiovascular risk stratification rather than longitudinal treatment response monitoring. Traditional assessment of statin response in carotid plaques relies on serial ultrasound or MRI, with most studies documenting plaque volume regression only after 12 months [25, 26]. Although one study using 3-T MRI demonstrated detectable changes in carotid plaque volume at six months [27], the sample size limits generalizability for clinical implementation.

Our CNN-LSTM temporal modeling approach enables early treatment response prediction at six months using widely available conventional ultrasound. The LSTM architecture identifies subtle temporal evolution trajectories across sequential multimodal imaging patterns that emerge earlier than conventional morphological thresholds detectable through traditional visual assessment. This capability reflects the fundamental advantage of temporal sequence modeling, which captures progressive changes in tissue characteristics across multiple imaging modalities rather than relying on isolated morphological measurements. The architectural choice aligns with recent advances in medical imaging, where hybrid CNN-LSTM models have demonstrated superior performance for longitudinal analysis requiring temporal dynamics assessment [28, 29].

### Model interpretability and feature contributions

The marked performance improvement from three to six months reflects the underlying biology of plaque stabilization. Statin-induced alterations progress through sequential stages beginning with fibrous cap thickening in early months, while lipid core reduction becomes detectable around six months [16, 30]. These sustained therapeutic effects manifest as consistent echogenicity enhancement and structural modifications across multimodal ultrasound, creating temporal evolution patterns that our CNN-LSTM architecture captures effectively.

Ablation studies confirmed the dominant contribution of longitudinal imaging features, with imaging-only models achieving performance closely approximating full hybrid models while clinical-only models showed substantially inferior results. Attention mapping analysis revealed selective focus on clinically relevant plaque regions across different imaging modalities, successfully identifying morphologically significant characteristics associated with treatment response [31]. This spatial attention capability offers clinicians visual guidance on high-risk regions, enhancing assessment beyond traditional subjective evaluation. Among clinical features, diabetes mellitus consistently emerged as the predominant predictor across temporal models, aligning with established mechanisms whereby diabetes impairs plaque stabilization through persistent inflammation and altered lipid metabolism [32].

### Limitations and future directions

Several limitations warrant acknowledgment. This study employed imaging-defined treatment response as the primary endpoint rather than clinical outcomes such as stroke or myocardial infarction. Imaging changes remain surrogate markers with inherent limitations in directly predicting clinical benefit. This single-center design utilizing a single ultrasound vendor limits generalizability, as different ultrasound systems vary in acquisition parameters, signal processing algorithms, and image quality characteristics, potentially affecting model performance in other clinical settings. The study specifically targeted patients with vulnerable plaques or severe stenosis, excluding the broader population with common atherosclerotic plaques and dyslipidemia who represent a significant portion of clinical practice, potentially limiting applicability to routine carotid atherosclerosis management. The inherent operator dependency of ultrasound imaging introduces potential variability that could affect model performance in real-world implementation despite standardized protocols.

Future research should prioritize external validation across multiple centers to confirm model generalizability and prospective trials testing whether model-guided therapeutic adjustment at 6 months improves cardiovascular outcomes compared to conventional management. Studies should systematically evaluate whether incorporating longitudinal lipid profiles or inflammatory markers at each follow-up time point enhances the 6-month prediction beyond imaging features alone. Expanding inclusion criteria to encompass patients with varying plaque severity and different lipid disorders would determine if the model requires recalibration for broader clinical populations. Development of standardized multimodal ultrasound acquisition protocols with quality control metrics would minimize operator-dependent variability, facilitating wider clinical implementation.

## Conclusion

This study successfully developed and validated a hybrid CNN-LSTM DL model integrating longitudinal multimodal ultrasound imaging with clinical data to predict carotid plaque treatment response to statin therapy. The model enables reliable response assessment within six months, substantially earlier than conventional methods. This capability could transform personalized treatment strategies by enabling timely therapeutic modifications for treatment-resistant patients.

## Supplementary information

ELECTRONIC SUPPLEMENTARY MATERIAL

## Data Availability

All data generated or analyzed during this study are included in this article. Further enquiries can be directed to the corresponding author.

## Abbreviations

AI: Artificial intelligence
AUC: Area under the curve
CNN: Convolutional neural network
DCA: Decision curve analysis
DL: Deep learning
FN: False negative
FP: False positive
HL: Hosmer–Lemeshow
LDL: Low-density lipoprotein
LSTM: Long short-term memory
MRI: Magnetic resonance imaging
ROC: Receiver operating characteristic
SMI: Superb microvascular imaging
SWE: Shear wave elastography
