# Supplementary material for: Deep learning-based early prediction of carotid plaque response to lipid-lowering therapy using longitudinal multimodal ultrasound imaging
Source: Insights Imaging. 2026 Apr 21;17:109. doi: 10.1186/s13244-026-02286-5 (PMC13100238; doi:10.1186/s13244-026-02286-5)
Supplement: Supplementary file 1 — ELECTRONIC SUPPLEMENTARY MATERIAL [file 13244_2026_2286_MOESM1_ESM.pdf]

Deep Learning-Based Early Prediction of Carotid Plaque Response to Lipid-Lowering Therapy Using Longitudinal Multimodal Ultrasound Imaging

ELECTRONIC SUPPLEMENTARY MATERIAL

Insights Imaging (2026) Jiang L, Sun Y, Huang W, et al.

Table S1. Comparison of baseline clinical characteristics between training and testing cohorts

Characteristic	Training Set (n = 637)	Testing Set (n = 165)	P-value
Age, years	64 (59, 69)	63 (59, 68)	0.086
Male gender, n (%)	372 (58.4)	100 (60.6)	0.608
BMI, kg/m²	26.2 (23.8, 28.4)	26.3 (23.4, 28.7)	0.606
Hypertension, n (%)	452 (71.0)	119 (72.1)	0.769
Diabetes mellitus, n (%)	224 (35.2)	54 (32.7)	0.558
Current smoking, n (%)	211 (33.1)	59 (35.8)	0.523
Family history of CAD, n (%)	149 (23.4)	38 (23.0)	0.922
Total cholesterol, mmol/L	5.3 (4.5, 6.1)	5.2 (4.5, 6.1)	0.669
LDL cholesterol, mmol/L	3.2 (2.5, 3.8)	3.3 (2.6, 4.0)	0.138
HDL cholesterol, mmol/L	1.3 (1.0, 1.5)	1.2 (1.0, 1.5)	0.734
Triglycerides, mmol/L	1.9 (1.4, 2.6)	1.8 (1.4, 2.3)	0.097
Antihypertensive therapy, n (%)	406 (63.7)	109 (66.1)	0.579
Previous statin use, n (%)	145 (22.8)	33 (20.0)	0.447

CAD: coronary artery disease; LDL: low-density lipoprotein; HDL: high-density lipoprotein.

Table S2. Training parameters and convergence metrics for deep learning models

Model	Best Epoch	Best Validation Loss	Best Validation Accuracy
baseline	23	0.583	0.674
3m	33	0.460	0.811
6m	100	0.115	0.977
9m	149	0.071	0.977
12m	87	0.113	0.955

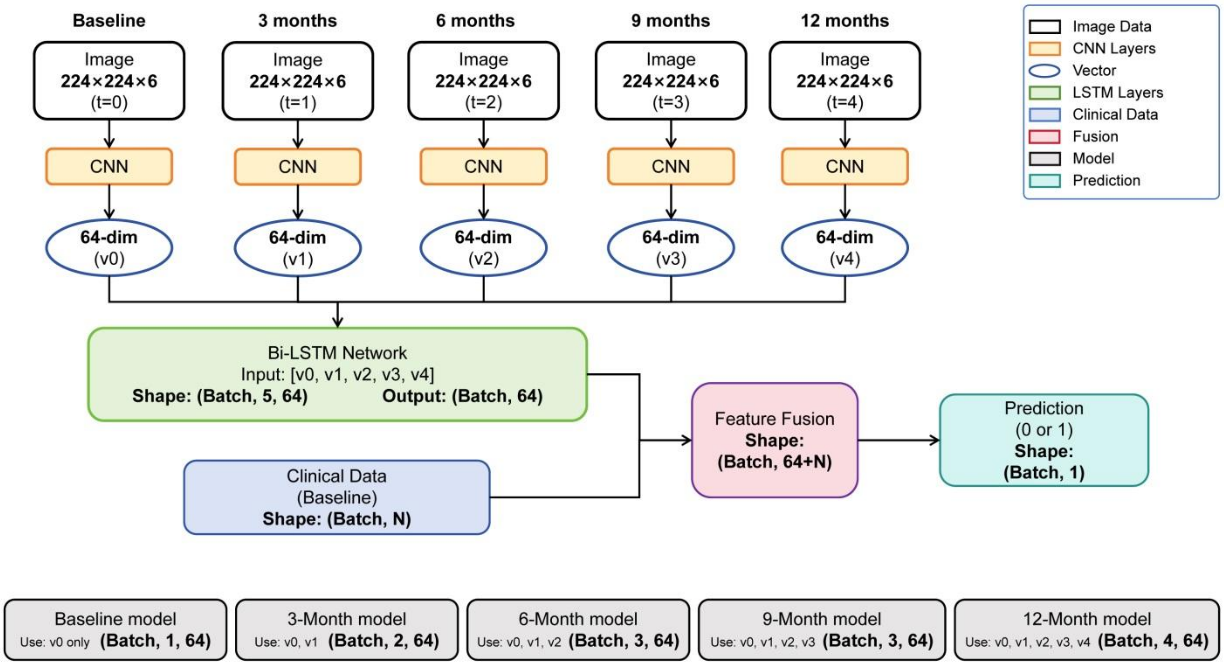

Figure S1. Data flow from raw images to final prediction. Batch refers to the number of patients processed simultaneously (16 during training). Clinical features (n=6) from baseline are processed separately and fused with imaging features. Each model used identical CNN-LSTM architecture but processes different length temporal sequences.
